# The meaningfulness of *exploring one's own limits* through interactions and enjoyment in outdoor high-intensity physiotherapy for people with multiple sclerosis: a qualitative study

**DOI:** 10.3389/fresc.2024.1303094

**Published:** 2024-03-18

**Authors:** Stine Susanne Haakonsen Dahl, Ellen Christin Arntzen, Britt Normann

**Affiliations:** ^1^ Faculty of Nursing and Health Sciences, Nord University, Bodø, Norway; ^2^ Department of Physiotherapy, Nordland Hospital Trust, Bodø, Norway

**Keywords:** physical activity, physiotherapy, multiple sclerosis, qualitative study, exercise therapy, postural balance, enactive theory

## Abstract

**Background and purpose:**

Physical activity (PA) is often reduced in people with MS (pwMS), even when disability is low. Understanding the perspectives of pwMS on interventions aiming to improve PA is important to inform the development of such services. The aim of this study was to explore the experiences of pwMS participating in an outdoor, high-intensity and balance exercise group intervention.

**Methods:**

This qualitative study was nested within an RCT exploring a novel intervention integrating sensorimotor exercises with high-intensity intervals of running/walking. Individual, in-depth interviews with the intervention group (*n *= 15; 12 women, 3 men; age 38–66; EDSS score 0–3.5) were conducted postintervention (mean days = 14), analyzed using a phenomenological-inspired approach with systematic text condensation, and interpreted based on enactive theory.

**Results:**

Four categories were generated: (1) *Exploration of one's own physical abilities:* Challenging one's own limits was perceived by all participants to improve movement performance and/or intensity level. Such bodily changes engendered strong positive feelings. Some negative consequences of high-intensity training were described, increasing a feeling of loss. (2) *New insights and beliefs:* Participants experienced enhanced beliefs in their own capabilities, which they integrated in activities outside the intervention. (3) *An engaging environment:* The group setting was perceived as supportive, and the outdoor environment was perceived as stimulating activity. (4) *Professional leadership, tailoring and co-creation of enjoyment:* Physiotherapist-led, individualized interactions were regarded as necessary to safely revisit prior activities, such as running. Co-creating enjoyment facilitated high-intensity training and intervention adherence.

**Discussion:**

High-intensity training combined with detailed exercises in a physiotherapy outdoor group was perceived to create meaningful bodily changes and enhance PA and prospects for both PA and life. Importantly, however, some negative experiences were also reported from the high-intensity training. Enactive theory allowed for the illumination of new perspectives: the importance of embodiment for self-efficacy and of tailored physiotherapy and an outdoor-group environment for exploring one's own limits to physical capabilities. These aspects should inform future exercise interventions in pwMS with low disability.

## 1 Introduction

Multiple sclerosis (MS) is a progressive inflammatory disease of the central nervous system (CNS) that is typically diagnosed at 30–40 years of age (1). A great concern is the significantly lower levels of physical activity (PA) in people with MS (pwMS) across disability levels than in their healthy counterparts (2, 3).

Early promotion of PA and exercise is recommended due to numerous established benefits in health, symptom management and well-being for pwMS (4). In particular, high-intensity training is endorsed, as it has possible neuroprotective effects in the disease course (5, 6). In addition, exercises addressing sensorimotor impairments (e.g., reduced muscle strength, reduced neuromuscular control) are recommended, as they target individuals' capability to remain physically active (7). Sensorimotor impairments can influence trunk control, which is commonly disturbed in pwMS, even when disability is low (8, 9), and correlate with impaired balance, walking capacity and distance (10, 11). PwMS's knowledge of exercise benefits, attitudes and motivations, as well as contextual aspects such as lack of optimal exercise interventions, accessibility and support, affect the level of PA and exercise participation (12).

CoreDISTparticipation (Table 1) is a new comprehensive intervention addressing sensorimotor function, trunk control, high-intensity running/walking and work participation in pwMS with low disability (13). It is based on the GroupCoreDIST^1^ intervention, which has been shown to have significant short- and long-term effects on trunk control, balance and walking among pwMS (14, 15). However, no effects of the intervention on objectively measured PA have been identified, even though the participants reported perceptions of new possibilities to be physically active as their sensorimotor impairments improved (16). To address PA challenges in pwMS, GroupCoreDIST was further developed to include a four-week period of outdoor training, in which high-intensity walking/running and GroupCoreDIST exercises are integrated (Table 2). To our knowledge, combinations of high-intensity training and rehabilitation of specific sensorimotor functions have been sparsely explored. Patient perspectives are essential for the evaluation of healthcare interventions (17); however, the new outdoor component of CoreDISTparticipation has yet to be investigated from a first-person perspective. Particularly interesting is what participants perceive as meaningful regarding the intervention, as this is essential for motivation, motor learning and exercise adherence (18).

**Table 1:** Overview of the CoreDISTparticipation intervention.

Week 1: MS outpatient clinic	*Consultation with the MS nurse* (20 min) to address work-related issues based on a structured guide comprising the following themes: knowledge of MS at the workplace, experienced work-related challenges due to MS, potential needs and facilitators**.**
	*Physiotherapy assessment* (60 min) to explore the potential for changes in balance and walking aiming to turn focus toward possibilities and thus, motivate the patient**.**
	Based on these assessments the MS nurse and the physiotherapist indicated the aspects of importance on a standardized form to inform the municipal physiotherapist.
	*Standardized testing* (baseline, for the RCT).
Week 2–5: Municipality	*Physiotherapy assessment* (60–90 min) to explore the patient’s impairments and potential for improvements in a clinical examination prior to group-training.
	*Indoor group* (60 min × 2 weekly, for 4 weeks). There were three to five participants in each group and one physiotherapist. Trunk control, balance and physical activity were addressed (GroupCoreDIST). Participants received a link to *CoreDIST digital exercise-videos* and were advised to do them 1 × weekly throughout the intervention. (videos can be accessed here: https://www.nord.no/en/node/35,098)
	*Digital meeting with a multidisciplinary team* (pwMS, employer, physiotherapist & MS nurse) (20 min) regarding barriers to work participation and needs for adaptations regarding work and physical activity, according to a structured meeting-guide (one meeting).
Week 6	*Standardized testing* (midway, for the RCT).
Week 7–10: Municipality	*Outdoor group* (60 min × 2 weekly, for 4 weeks). A maximum of ten participants and two physiotherapists were included in each group. Trunk control and balance (GroupCoreDIST exercises) were addressed, and high-intensity walking or running was performed. The intervention was conducted in a city park where both flat and uneven surfaces and hilly terrain were available (Table 2).
	Additionally, participants were encouraged to comply with the exercise-videos through a weekly SMS-reminder.
Week 11–14	*Standardized testing* (final, for the RCT) and *qualitative interviews.*

**Table 2:** Description of the outdoor group.

Content	Purpose
Warm-up and recording one’s own balance	
Exercises for detailed sensorimotor activation, larger muscle groups, muscle length and balance while standing.	Preparation.
	Experience one’s own balance and record eventual changes.
Dual task: motor (using spiky balls and medicine balls individually, in pairs and in the group) and cognitive (singing, rhymes and counting).	
Main part	
(1) High-intensity training (85%–95% maxHR/min 16 RPE) × 4 min: Running or walking with long strides and large arm movements. Participants chose their own route, marking it with a cone, and picked up a bean bag for each new lap to count how many laps for each interval.	Improve stamina.
	Experience one’s own opportunities for high-intensity physical activity.
	Improve sensorimotor control and balance as prerequisites for walking and running.
(2) Moderate-intensity detailed exercises (approx. 70% maxHR) × 3 min. CoreDIST exercises while standing approximately (10 repetitions × 2 set). Examples of exercises: squat, one legged stance, rise on toes, reaching, turning and rolling down to touch the ground in standing.	
Progressions was individually tailored (during both running/walking and the detailed exercises) through instructions, demonstration and hands-on facilitations by the physiotherapists. Quality and efficiency of movement were addressed by the physiotherapists. Optimalization of trunk control during movement were emphasised.	
A combination of high-intensity and CoreDIST exercises was repeated 3–4 times during one session.	
Cool-down and recording one’s own balance	
Hold/relax muscle contraction.	Experience one’s own balance and record eventual changes.
Balance on one leg.	

To deepen our understanding of what the participants perceive as meaningful, we turn to a theoretical perspective that integrates bodily capacities with the construction of meaning. Enactive theory emphasizes that making sense of the world depends essentially on the biological (living) body and the phenomenological (lived or experienced) body (19), which implies that the body is viewed as a neurobiological organism that is concurrently experiencing, expressing and social (embodiment) (20). Thus, what is experienced by an individual during an exercise intervention is constituted by her sensorimotor repertoire for perception and action in interactions with the requirements of the task and the context (21). From this perspective, dysfunctions related to MS, such as sensorimotor impairments, can influence how individuals with MS interpret and understand their participation in a PA intervention. Moreover, the notion of “participatory sense-making” (22) extends the body into the social domain, enabling an understanding of how the interaction processes between two embodied individuals affect shared and individual meaning-making. These concepts may illuminate pwMS's experiences and direct the focus toward bodily, contextual, and interactional aspects that may generate new insights regarding sensorimotor exercise and high-intensity training as part of PA.

The aim of this study was to explore participants' experiences of the content, delivery and setting of a new outdoor group intervention combining high-intensity training and detailed exercises to generate new knowledge about important aspects of exercise interventions for pwMS with low disability.

## 2 Materials and methods

### 2.1 Design

Individual in-depth interviews using a phenomenological-inspired approach were chosen, as this is suitable for exploring the meaning and significance of pwMS's experiences and reflections (23, 24).

### 2.2 Ethical considerations

The study was conducted according to the Declaration of Helsinki and approved by the Regional Committee for Medical Research Ethics in North Norway (REK North: 174837). Written informed consent was obtained prior to the intervention and confirmed verbally when arranging the interviews. Participation was voluntary and anonymous, and the participants were informed about the opportunity to withdraw from the study. The Consolidated Criteria for Reporting Qualitative Research (COREQ) (25) were used to optimize the conduct and reporting of the study.

### 2.3 Study context

This interview study was nested within a randomized controlled trial (RCT) comparing the CoreDISTparticipation intervention to usual care (26) and conducted at a regional hospital MS-outpatient clinic (Nordland Hospital Trust) and in two affiliated municipalities in the northern Norway. The current study investigates participants in the intervention group's experiences of the four-week outdoor group, which was part of this new intervention (Table 2). The outdoor sessions were conducted by three trained physiotherapists working in the community healthcare in the two municipalities. The project team included three individuals representing users from the Nordland MS Association, along with an MS nurse and a neurologist from the MS-outpatient clinic, and three physiotherapists/ researchers.

### 2.4 Research team and reflexivity

All researchers on the team are clinical specialists in neurological physiotherapy. BN and ECA developed the CoreDISTparticipation intervention, and SSHD contributed to the development of the outdoor part.

The researchers' closeness to the intervention and the clinical field may have strengthened the depth and relevance of their interpretations in this study (27), as it was easy to understand what participants described and helped form follow-up questions during the interviews. However, closeness may also produce a risk of “blind spots”, as the researchers may prejudice participants' experiences, omitting questions where the answers are believed to be obvious (27). Thus, throughout the process, trustworthiness and rigor were enhanced by discussing the methodology, findings, and interpretations with external researchers (including specialists in enactive theory), as well as user representatives. The presented theoretical framework (enactive theory) enhanced the distance to the material, as recommended in qualitative research (28).

### 2.5 Recruitment and participants

Prior to recruitment, the study was introduced to individuals with multiple sclerosis (pwMS) through a seminar hosted by the Nordland MS Association. Additionally, seminars were conducted for health professionals in community healthcare and at the regional hospital. Written information about this study (and the RCT) was sent from the MS clinic at the regional hospital by post to all eligible individuals affiliated with the hospital. Individuals who wished to participate signed the attached consent form and returned it in the pre-stamped envelope. The inclusion criteria were as follows: had been diagnosed with MS, had a score on the Expanded Disability Status Scale (EDSS) (29) of ≤3.5, was ≥18 years, was employed (10%–100% of full-time) and residential address in the two predefined municipalities. The exclusion criteria were as follows: pregnancy, exacerbation of symptoms within two weeks prior to enrollment and other serious conditions compromising balance, walking or work capacity. All participants in the intervention group of the RCT (*n *= 15) were included (Table 3).

**Table 3:** Participant demographic information.

Variable	Total (*n *= 15)
Age in years	Mean 47.6 (SD 6.04)
Gender (women/men)	12 woman/3 men (80%/20%)
Type of MS	Relapsing remitting 15 (100%)
EDSS	Mean 1.8 (SD 0.9)
Years since diagnosis	Mean 10.4 (SD 7.8)
Participation in the outdoor group	Mean 4.6 sessions/total mean attendance 57.3%

### 2.6 Data collection

The interview guide (Table 4) was developed based on literature reviews, clinical experience and discussions within the research group and with user representatives. Two test interviews were conducted (with pwMS who were not part of the sample), and the interview guide was then refined around the following themes: overall experience and reflections from participation, content, outdoor setting, the group, and the physiotherapists. Questions were open-ended to capture rich, in-depth reflections regarding participants' experiences, following a phenomenological approach. The interviewer asked for both negative and positive experiences and rephrased and asked follow-up questions to clarify and confirm the correct understanding of participants' answers.

**Table 4:** Interview guide.

Theme	Potential questions
Overall experiences and reflections from participation	Generally, what are your main experiences of participation?
	What did you perceive as meaningful?
	What did you perceive as negative?
Content	How did you experience:
	• The content of the sessions in general • The high-intensity walking/running • The specific exercises • The combination of specific exercises and intervals of running/walking • The exercise intensity
	How did you respond to the exercises? How did you experience getting tired?
	How do you perceive your specific movement impairments (if any) being addressed?
	Please elaborate on situations where you experienced the feeling of mastery/failure.
	If anything: What was challenging? What would you prefer to have been done differently? What did you enjoy?
	What was the value of participating in the indoor exercise group beforehand?
	How did you experience this kind of exercise intervention compared to other type of exercise you may have experience with?
The role of the physiotherapists	What did the physiotherapists do? What was the value of this to you?
The group setting	How did you experience the group setting?
	How did you perceive the atmosphere in the group?
The outdoor environment	How was it to exercise outdoors?
	How did you perceive the city park environment for exercise?
Closing questions	Are there any experiences from participation that you would like to elaborate on? Is anything related to this project that we have not talked about that you would like to say?
	How did you experience this interview?

Overall participants were asked to describe situations to exemplify their answers, and follow-up questions were used to capture in-depth reflections, for example, *What was positive/negative?, How did it feel?, What do you think of that?, What does it mean to you?, Can you elaborate on that?*.

As similar themes arose repeatedly and no new themes emerged in the final interviews, data saturation was achieved (23).

### 2.7 Analysis

**Figure 1:**
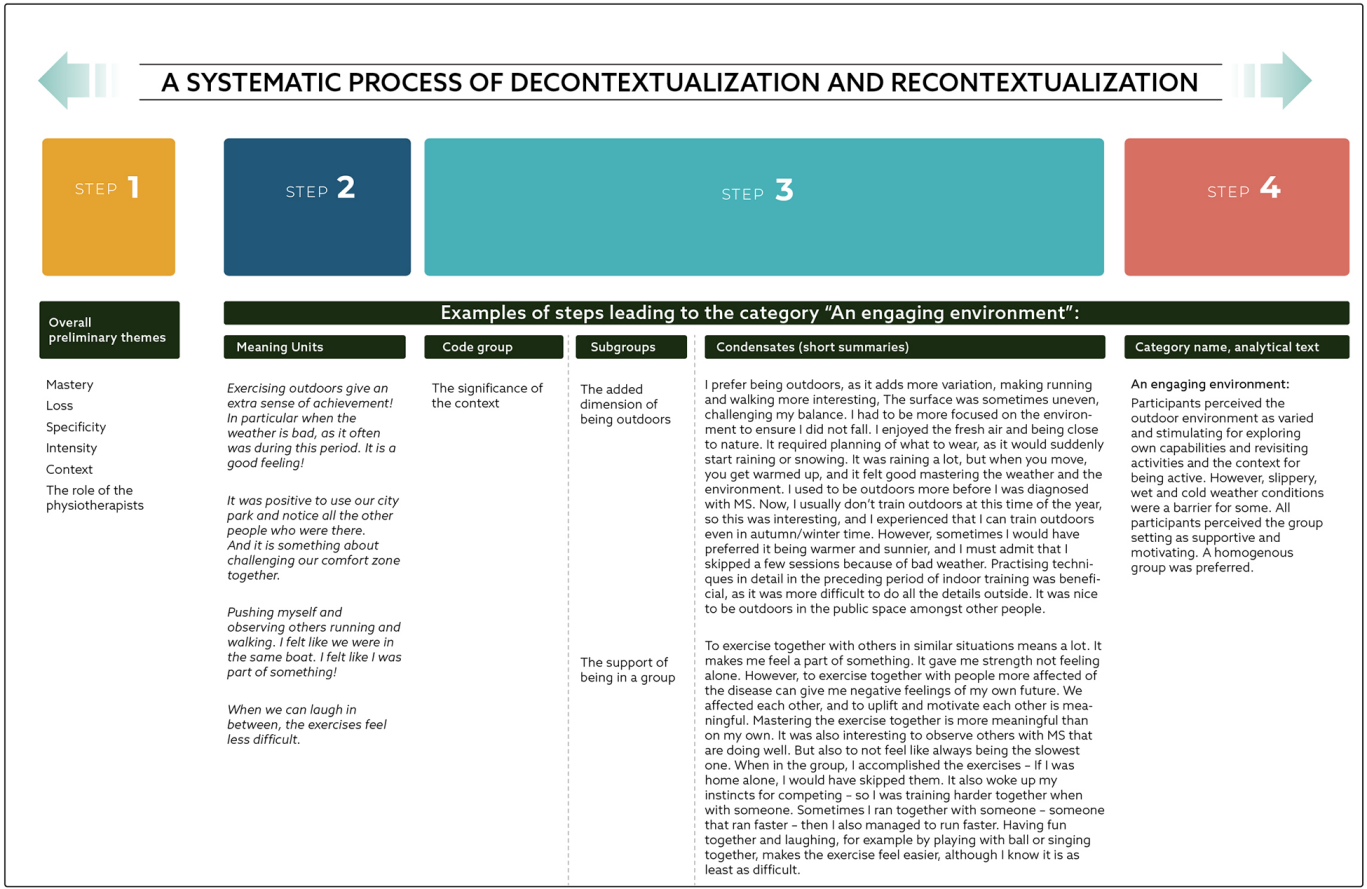
Example of the analysis process (excerpts).

The transcribed material was analyzed using systematic text condensation (STC) (30) and was organized utilizing NVivo (version 1.7.1). STC is a method for cross-case analysis inspired by phenomenology. It involves four-steps: (1) identification overall themes from the empirical material, (2) extraction of meaning units from the text which were then coded into groups, (3) condensation of all meaning units within the subgroups into an artificial quotation, that summarize and represents participants' voices, (4) recontextualization of the material into categories, presented as analytical texts. The process is iterative, resulting in continuous movement between the transcripts and within different steps of the analysis. An example of the STC process is illustrated in Figure 1.

**Figure 2:**
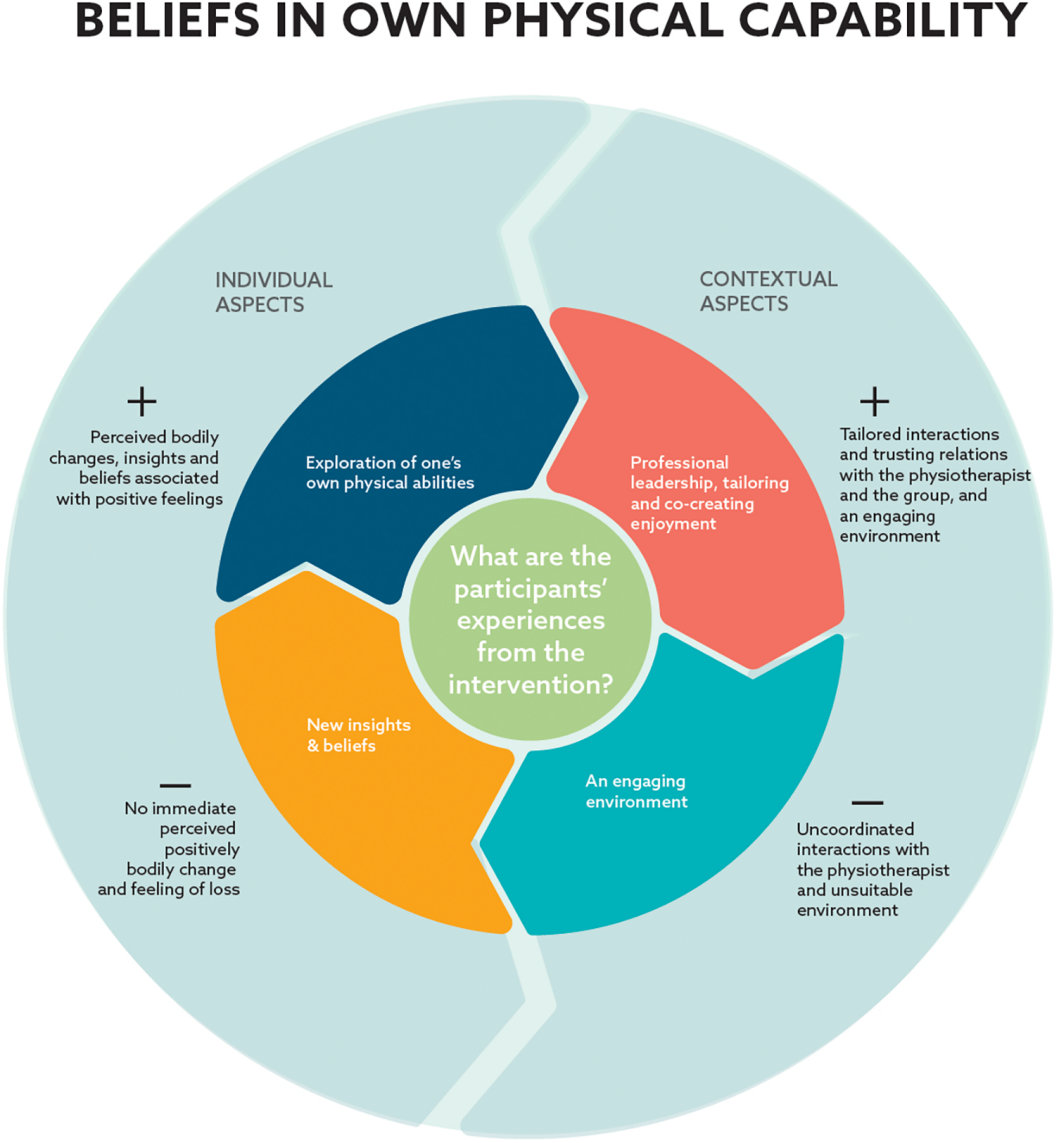
Model of participants’ experiences and interconnections. The figure displays the four categories generated (inner circle). The categories *Exploration of one's own physical abilities* and *New insights & beliefs* represent individual aspects, and *Professional leadership, tailoring & co-creation of enjoyment* and *An engaging environment* represent contextual aspects shaping participants experiences. Individual and contextual experiences from the intervention were perceived by participants as promoting (+) and hindering (−) their beliefs in own physical capability (outer circle).

The first author (SSHD) transcribed the interviews and read all material several times, while BN and ECA read most of the interviews before preliminary themes were agreed on. SSHD identified meaning units adhering to these themes and coded them into groups. Condensates of the subgroups were written by SSHD and discussed by all researchers. SSHD then recontextualized the material by forming categories described as analytical texts supplemented by quotes, a process that was discussed and revised several times by all authors. All authors contributed to writing the manuscript. Enactive theory was used to interpret the results, aiming at extracting new knowledge beyond what the informants had provided (28).

## 3 Results

Participants were interviewed one-on-one by the first author (SSHD) in November and December 2021 (mean = 14 days post-outdoor group). The time and place of the interviews were agreed upon according to participants' preferences (undisturbed office (*n *= 14), participant's home (*n *= 1)). None dropped out. The interviews lasted between 40 and 70 min (mean = 54, total = 822) and were audio-recorded.

The results are presented as four categories summarized in Figure 2 and described below as analytic texts and illustrative quotes referenced with the participant ID and EDSS score.

### 3.1 Exploration of one's own physical abilities

Overall, the participants reported strong positive experiences of being supported and pushed to explore the limits of their physical abilities. All participants reported benefits from these explorations, such as improved performance in the exercises, higher training intensity or functional gains such as improved balance or the ability to run again. The organization of the intervals of the exercises supported participants' ability to increase their training intensity. Participants felt that the detailed exercises in between intervals made them more ready or warmed up for the next running/walking interval. The opportunity to choose the terrain (hilly or flat) and walking/running route were appreciated, and technique corrections by the physiotherapist were reported to facilitate physical performance. The majority of participants highlighted that they reached higher intensity than expected during these sessions, and for many, the outdoor intervals mediated participants' strong emotional experiences. One person described this as follows:

*The feeling of using my body! That my legs are working and I can run! It is a positive feeling—enhancing the feeling of being alive! I also enjoyed feeling tired after training—as it gave my negative thoughts a break.* (ID10, EDSS: 1)

However, two individuals reported a soft-tissue injury during running (ankle sprain, hamstring strain), one experienced increase in dizziness, and three described negative feelings associated with their failure to achieve high intensity:

*It was somewhat distressing during the last interval, as my feet were not working, and I did not know what to do to increase my heart rate—when I could not run or walk quickly anymore.* (ID2, EDSS: 2)

The focus on the core or the middle of the body in the detailed exercises was stated to improve participants' PA performance; participants described being less clumsy or unsteady or walking without holding on to the walls. Having practiced the detailed CoreDIST exercises in the indoor group beforehand was described as a helpful and pertinent preparation by some participants, as it was regarded as more difficult to accurately execute the exercise techniques outdoors due to their higher intensity, the uneven surface, or bad weather. Some participants commented that the standing exercises (in-between the running/walking intervals) required too much effort, leaving their legs tired for running afterward.

### 3.2 New insights and beliefs

A key feature of the participants' stories was their new insights into their own physical abilities, which were perceived to influence their beliefs about their own possibilities for PA and life in general:

*What meant the most for me was the high-pulse training, as I had thoughts of it being a left behind phase for me. The experience of being able to master it felt so good. It enhances my focus on future possibilities rather than limitations.* (ID4, EDSS: 0)

Gains in insight were also reported from the detailed exercise part of the sessions, highlighting how the function of body parts through movements and sensations was linked to performance in PA, as illustrated below:

*I have simply been taught some tools to improve certain parts of my body and how that has an effect on, for example, walking: That my hip has to be with me to maintain balance—and that makes how I stand on the ground important. Previously I was not aware of that…., now everything works better.* (ID6, EDSS: 2)

Two participants reported that the intervention motivated them to commit to new exercise routines, and some stated that they had more “readiness” for activities such as playing with their grandchildren, hiking with friends, or engaging in a high-intensity activity. Some stated that their bodily changes were perhaps not noticeable for others, but they themselves noticed that it was easier to climb stairs, balance on one leg and walk fast or that they now moved in a “better way” or with less pain. Three participants perceived the duration of the outdoor group to be too short to feel lasting improvements in their physical endurance or muscular strength.

### 3.3 An engaging environment

Most participants reported that their performances were positively influenced and motivated by the group setting, for example, through cooperating in exercises with balls, seeing other individuals in the group who were “doing well”, cheering each other and competing when running and walking next to each other. However, one participant emphasized that observing people with visible disabilities from MS was distressing, as it revealed negative thoughts about one's own future. It was emphasized that mastering challenges in the group sessions added more meaning than doing the same alone:

*I think this particular exercise is hard work, and then it becomes very tiring to do it on my own. However, when I did it in the group and we could laugh a bit in between and so on, it was easier because of the social element.* (ID12, EDSS: 1.5)

Being active outdoors was preferred by many participants because of the fresh air and the natural and varied environment:

*It was an added positive experience to use our city park and notice all the other people who were there…it is something about challenging our comfort-zone*. (ID4, EDSS: 0)

The natural environment was also described as taking focus away from MS symptoms. Cold, rainy or snowy weather conditions required planning of adequate clothing; in addition, these conditions led some participants to use cautious behavior when the ground was slippery and led a few to omit sessions. However, mastering outdoor exercise was highlighted in positive terms, such as discovering new ways to become active.

### 3.4 Professional leadership, tailoring and co-creation of enjoyment

The way the physiotherapists led the group and, in particular, interacted with each participant were regarded as helpful for improving their bodily functions and activity levels. Some participants reported being afraid to try out new activities or training at high intensities after being diagnosed with MS but felt safe to explore when supervised by the physiotherapist because of their trust in the relationship between them and in the physiotherapist's professional knowledge.

How the physiotherapist approached the participants individually was described as important from this perspective. In particular, bodily interactions in which the physiotherapist demonstrated with his or her own body or placed his or her hands on the participant's body to correct a movement were reported to be successful, as it helped to increase speed and gave participants a sense of performing better or for a longer duration. If they did an exercise in a suboptimal way, participants reported receiving precise supervision, or if they expressed pain or were injured, the physiotherapist was supportive, assessed them and gave them advice for follow-up. Some participants said that when the physiotherapist conducted the exercises or ran/walked together with them, it made them increase their exercise intensity. One participant described this as follows:

*The physiotherapists pushed me to perform beyond what I thought I was able to—and that was great! There is no doubt that if someone is running beside you and shouting “come on- well done”, you manage to push yourself further.* (ID8, EDSS: 2)

However, one participant described an incident where the interaction with the physiotherapists was not perceived as helpful:

*When I get tired, it gets difficult. I can only do one thing at a time, and then these physiotherapists came running, talking and trying to motivate at the same time. I got very tired, and my leg would not follow my commands to run.* (ID7, EDSS: 3.5)

Participants reported that they appreciated that the physiotherapists made them engage in playful activities with a ball, run for beanbags, and sing and in general created an informal and nice atmosphere. The enjoyment created was described as important for adherence to the intervention and as encouraging participants' physical effort during the session, as exercise felt easier when it was enjoyable. It was appreciated that the physiotherapists were perceived as both cheerful and serious about the intervention.

## 4 Discussion

The main findings of this study are that (1) being supported to explore and push one's own physical capabilities by combining high-intensity running/walking with detailed exercises was meaningful and evoked strong emotions. Improving one's balance, walking, and running lead to increased beliefs in one's own possibilities. Some negative experiences were also described, particularly from the high-intensity training. (2) An engaging outdoor group with tailored physiotherapist-participant interactions and the co-creation of enjoyment was perceived to be important for the success of the individual. These findings illustrate how the dynamic intertwining of the body and movement, context and intersubjective interactions create meaning and beliefs in one's own physical capabilities (19).

### 4.1 Bodily experiences are inherent to beliefs in the mastery of physical activity

The meaningfulness of exploring the limits of training intensity that we identified in our study corresponds with other studies of pwMS's experiences of interventions addressing intensity of activity (31, 32). The exercises emphasizing trunk control were reported to reduce movement impairments and are in line with a study of pwMS with higher disabilities participating in an indoor group intervention (16). However, the perceived interlinking of improved sensorimotor functions and the ease of and efficiency in high-intensity walking/running have not been reported previously. It is likely that the detailed exercises prompted activations of the CNS and musculoskeletal systems, which are prerequisites for high-intensity walking and running (33). Impairments in such systems commonly occur due to CNS lesions or secondary inactivity, and function can improve with increased use (18). Our results support the value of integrating such specificity to optimize the capability to train at high intensity, even in individuals with low EDSS scores.

The described emotional associations of these bodily changes are interesting. Achieving higher exercise intensities, easier movements, reduced pain and improved sensation lead to positive feelings and enhanced prospects for both PA and life, while for some individuals, a failure to achieve high-intensity or no immediate changes in impairments are associated with feelings of loss and negative prospects. This calls attention to acknowledging that sensorimotor capacities facilitate or constrain *how* an individual perceives the world, which is closely interlinked with feelings, and that influence *why* participants perceive what they do (34). These experiences necessitate that sensorimotor changes in pwMS involve not only their biological body but also their relational and self-individuating modes of operating in the world, including how an experience coheres with, for example, participants' historical experiences (35). As we primarily regulate such modes to achieve an optimal positive mood state, this can also explain why only changes perceived as positive appear to enhance participants' beliefs for the future (36). Negative experiences such as failure to achieve high intensity because the legs are not working in the last interval can thus be perceived as detrimental by pwMS.

We argue that participants' perceived bodily changes affected their self-efficacy for being physically active. Self-efficacy involves an individual's perception of exerting control over his or her own actions (37) and has been extensively reported to be pertinent to PA engagement in pwMS (38, 39). However, self-efficacy is theoretically described according to social cognitive theory (38). Our findings highlight how experiencing, expressing and socially interacting through the body (embodied experiences) shape individuals' self-efficacy and suggest a crucial role of bodily perceptions in constituting self-efficacy for PA.

### 4.2 Interactions and environment shape meaning making

Participants perceived the group setting to increase motivation, support, and commitment, which has been found in previously published work (16, 31).

The physiotherapist-participant interaction is acknowledged in exercise interventions for pwMS, pointing to professionals' role in informing participants of exercise benefits in the management of MS, including the prescribing mode, frequency, intensity, and duration of exercise (40). Tailored interventions are supported given the heterogenic pathology and symptoms of MS (41, 42). However, our findings illuminate qualitative aspects of how to achieve tailored and meaningful intersubjective interactions in an exercise intervention.

We consider the instances of the physiotherapist running together with the participant, which were perceived as important for participants' performance, to be an example of “participatory sense-making” (22)*.* As participants appreciated being guided or even pushed by the physiotherapists, it appears that the physiotherapists were trusted in directing this interaction. As such, we argue that the physiotherapists' ability to adapt to participants' movements, speech and gestures—tailoring the interaction to their needs—was important for this ability to be perceived as purposeful. This is supported by the few negative incidents described where the participant-physiotherapist interaction seemed to not be jointly coordinated and appeared to fail. The reported mutual influences of sensorimotor capabilities and interpersonal coordination, with the physiotherapists but also the group, are in accordance with sensorimotor capacities and intersubjective interactions being important for sense-making in the world (35). The benefits of these individualized participant-physiotherapist interactions are also described in specific core-stability exercises in indoor groups (16, 43) and are in line with the theoretical framework of facilitation of movement through hands-on interaction previously proposed (44, 45). Our study informs new knowledge of physiotherapist-participant interactions to achieve the recommended high-intensity training and calls for physiotherapy clinical reasoning through bodily and verbal communication skills adapted to the participants' responses in an ongoing and situated way.

Enjoyment has previously been reported to promote PA in pwMS, and our study brings requested knowledge of what can constitute enjoyment in an exercise intervention (46): playful group-exercise tasks, a cheerful physiotherapist, and the outdoor environment.

The appreciation of being active outdoors in the study sample aligns with that in the general population (47). The outdoors provided a natural environment, which both invited participants to actively explore abilities thought of as left behind after their diagnosis with MS, such as running, and provided an appreciated break from focusing on MS symptoms. We also suggest that the positive experiences of mastering the challenging weather conditions and the added meaning of exercising among other people in the city park can be explained according to such terms. These positive experiences show how we are enmeshed in our history, context and social encounters (35) and how these aspects should also be accounted for when designing exercise interventions.

### 4.3 Methodological considerations

The design and methods were adequate for deriving knowledge from individuals' experiences. The participants self-referred to the intervention and were recruited based on pre-set criteria. This approach yielded rich information from people with mild to moderate disabilities due to MS who were motivated for physical activity (PA), employed, and residing in northern Norway. Ethnicity or socio-economic class were not recorded. However, considering that all these factors can influence PA engagement (46), it is possible that additional aspects of the phenomenon could be uncovered in a different sample (48). There was a higher percentage of women participating than men; however, this corresponds to the gender distribution in the MS population (1).

The use of enactive theory was innovative within the field and allowed for, in particular, new aspects of importance for self-efficacy to be identified. Transference of our results to similar populations can be achieved through theoretical generalization (28).

### 4.4 Implications for clinical practice

Combining high-intensity walking/running and detailed sensorimotor exercises was valued and provided meaningful embodied experiences, improving participants' ability to master PA and their beliefs of their own possibilities for being active in the future. However, the manner in which the content of an exercise intervention is delivered and the environment in which it is delivered should be accounted for, as these aspects were perceived to be of great importance in creating and shaping participants' experiences. In particular, tailored physiotherapist-participant bodily interactions and an engaging group and outdoor environment were perceived to be pertinent for exploring one's own potential.

To minimize negative incidents in future interventions, we suggest that (1) the effort required from one's leg muscles during the detailed exercises (in between the running/walking intervals) should be low to minimize the negative consequences of leg muscle fatigue prior to high-intensity running/walking, (2) the capacity for running/walking at high-intensity should be explored in one-to-one physiotherapy assessment prior to group training to optimize individuals capabilities and safety, and (3) homogenous and small-sized groups should be used to enable ongoing and tailored physiotherapist-participant interactions.

## Data Availability

The datasets presented in this article are not readily available because of ethical and legal restrictions. Requests to access the datasets should be directed to stine.s.dahl@nord.no.

## Footnotes

^1^GroupCoreDIST is a group-based intervention (Group), involving 35 exercises at different levels, addressing activation of trunk musculature (Core) in motor tasks in lying, sitting and standing (e.g. rolling, reaching, squatting, single leg stance. DIST describes essential elements of the concept: D = dose (high), dual task; I = individualization, insight, intensity; S = sensorimotor activation, selective movement control; T = task oriented training.
